# Cervical Cancer Screening for the Reluctant - HPV Testing of Air-Dried Vaginal Discharge

**Published:** 2006-12

**Authors:** Tommy R. Tong, Rae Wai-nang Yau, Olivia Wai-hing Chan, Vivian Yu, Joyce Wing-sze Lo, Tat-chong Chow, Billy Wai-hon Chan, Kam-cheong Lee

**Affiliations:** 1 *Departments of Pathology, Princess Margaret Hospital, Hong Kong SAR, P. R. China;*; 2 *Department of Pathology, Alice Ho Miu-ling Nethersole Hospital, Hong Kong SAR, P. R. China;*; 3 *Obstetrics and Gynecology, Princess Margaret Hospital, Hong Kong SAR, P. R. China;*; 4 *Medical Gene Center Limited, Hong Kong SAR, P. R. China*

**Keywords:** human papillomavirus, HPV, cervical cancer, cervical intraepithelial neoplasia, air-dry, vaginal discharge, self-collect

## Abstract

Despite the availability of the PAP test, cervical cancer continues to cause considerable morbidity and mortality. Many women default cervical cytology for a variety of reasons. This demands the development of alternative screening strategies, such as HPV testing on self-procured cervical-vaginal specimens in order to capture this group of women. We investigated the self-procured air-dried vaginal discharge for HPV testing. We recruited 82 patients with HPV-associated cervical lesions and 36 patients with normal cervical pathology. Participants were briefed and informed consents obtained. Each was then given a kit containing written instructions, a slim napkin, an empty zip-lock plastic bag for soiled napkin specimen, and a return envelope. After wearing the napkin for the day, the patient removes it, dries it, and returns the specimen by mail. Specimens were batched and a 0.5 cm area of each stained napkin was tested for HPV by PCR. Specimens from all 26 patients with high-grade (CIN 2 or above) HPV-induced cervical lesions and 4 of 36 normal subjects tested positive for HPV, giving a sensitivity and specificity of 100% and 88.9%, respectively. We propose offering to women who refuse cervical cytology the alternative screening strategy of testing of self-procured air-dried vaginal discharge for HPV. This method of cervical cancer screening is also suitable for people living in remote regions of the world.

## INTRODUCTION

For half a century, the PAP smear was the only practicable way to screen for cervical pre-neoplasias and neoplasias. However, the inadequacies of the PAP smear is well known to practitioners of cytology (1, 2). It is also expensive both to patients and providers. A recent study in the United Kingdom has estimated the cost per life saved from premature death from cervical cancer at £36,000 (3). In addition, women are finding it increasingly difficult to find time for cervical cytology. This results in many cervical cancers occurring in women who have never had the PAP test. In Hong Kong, up to 50% of women do not participate in cervical cancer screening. Worldwide, this translates into considerable numbers of women being at risk for premature deaths from cervical cancer.

High-risk HPV is found in almost all cervical cancers. With the discovery of the necessary role of human papillomavirus (HPV) in cervical carcinogenesis (4, 5), HPV nucleic acid testing has been advocated to triage difficult cytology cases (6, 7) and vice versa (8). Recently, the Food and Drug Administration approved the use of HPV testing for cervical cancer screening in association with the PAP test in women over the age of 30 (9, 10). Because HPV testing as a means of cervical cancer screening is a more economical alternative for developing countries (11, 12), the question now being asked is whether HPV testing can replace the PAP test in cervical cancer screening (2, 13).

There are considerable interests in exploring alternative specimens and methods for their collection for the diagnosis of HPV-induced cervical lesions (HCL). Thus, urine, vaginal tampons, and other means of self-procurement of cervical cells have been tested, with various successes and limitations (14-18).

In 1869 Friedrich Miescher discovered DNA from pus that had collected on soiled surgical bandages (19). Recently, we showed that HPV DNA could be recovered from soiled sanitary napkins of women with cervical HPV infection (20). In this study, we aimed to validate HPV testing of self-procured air-dried vaginal discharge for the screening for significant HCL. We also determined the value of such specimens in predicting the results of surgical ablation of HCL.

## METHODS

### Patients

Patients referred to our hospital with abnormal PAP smears (atypical squamous cells of undetermined significance (ASC-US) or above) were screened by clinical history, repeat PAP smear, and colposcopy, with or without biopsy. During follow-up visits, patients with confirmed HCL (Table 1) were invited to participate in the study.

**Table 1:** Categories of HPV-induced cervical lesions

Pathology	Number of cases

Condyloma and CIN 1	54
CIN 2 and CIN 3	16
Squamous carcinoma	8 (1 microinvasive)
Adenocarcinoma in-situ	2
Endocervical adenocarcinoma	1
Clear cell carcinoma of cervix	1

CIN 1, cervical intraepithelial neoplasia grade 1; CIN 2, cervical intraepithelial neoplasia grade 2; CIN 3, cervical intraepithelial neoplasia grade 3.

To determine the value of HPV testing in patients who underwent ablative surgery for HCL, we recruited nine patients at 6 week after surgery for cervical intraepithelial neoplasia (CIN) by loop electrosurgical excision procedure (LEEP).

The study was approved by the Clinical Research Ethics Committee of Princess Margaret Hospital, Hong Kong. Participants were briefed and consent obtained. Patients were then given a kit containing the following items: demographics and registration form, written instructions, commercially available slim sanitary napkin, zip-lock plastic bag, and return envelope. Patients were instructed to use the napkin on any day between periods. The napkin was introduced in the morning. At the end of the day, the sanitary napkin was removed, placed inside the zip-lock bag and gently blown dry. The specimen was then mailed to our laboratory for testing. We collected all the specimens and stored them in the laboratory at room temperature until testing. When all specimens have been collected, we accessioned the specimens and commenced batch testing.

### Specimen Processing

Researchers are blinded to the pathology results. Care was taken when handling specimens to prevent cross-contamination. All specimens were opened in the biological safety cabinet and visually inspected for stains. A 0.5 cm piece of napkin containing stains was cut out from each specimen and inserted into an Eppendorf tube for DNA extraction. Gloves were changed between cases. As a check against contamination, we intercalated an unused napkin for every ten specimens.

### Molecular Methodology

DNA extraction was carried out by the addition of 600 ul cell lysis solution (Puregene DNA isolation kit, Gentra Systems, USA) and 3 ul proteinase K (20 mg/ml). The specimen was vortexed and incubated overnight at 56°C. After cooling to room temperature, the napkin fragments were removed. 200 ul protein precipitation solution was added to the cell lysate and the tube vortexed vigorously at high speed for 20 seconds. Centrifugation was carried out at 14,000 rpm for 5 minutes to pellet the precipitated proteins. This step was repeated if the pellet was not tight. The supernatant containing extracted DNA was pipetted into a clean 1.5 ml microfuge tube containing 600 ul 100% isopropanol. One ul of 20 mg/ml glycogen solution (DNA carrier) was added. The tube was inverted 50 times to mix the sample thoroughly, followed by centrifugation at 14,000 rpm for 2 minutes to pellet the DNA. After pipetting off the supernatant as far as possible, 300 ul 70% ethanol was added and the tube inverted several times to wash the DNA pellet. This was followed by centrifugation at 14,000 rpm for 5 minutes and the pipetting and discarding of the supernatant. The pellet was then air-dried for 15 minutes with the cap open. This was followed by the addition of 30 ul of DNA hydration solution.

The specimen was then aliquoted into two separate reaction tubes for PCR, using GP5+/GP6+ primers for one tube and MY09/MY11 for the other tube as previously described (21, 22). Negative controls consisted of water and unused napkin controls. Positivity on either reaction tube was considered conclusive for the presence of HPV.

Selected cases were typed by DNA sequencing and/or flow-through hybridization (HybriMax™; HybriBio limited, Hong Kong).

## RESULTS

Eighty-two patients with HCL were recruited. They represented a cross section of lesions ranging from condyloma to invasive squamous carcinoma, adenocarcinoma and clear cell carcinoma of cervix (Table 1). Visible yellowish to brownish stains were present in all used napkins.

All specimens with CIN 2 or above, with the exception of a small cell carcinoma tested positive in at least one PCR reaction (Table 2). We then tested the small cell carcinoma biopsy and obtained negative results by both PCR protocols. Excluding the case of small cell carcinoma, the sensitivity of dual-PCR tests on napkin specimens for CIN 2 or above was 100% (26 of 26 cases). The sensitivity for lower grade lesions were as tabulated (Table 2). The specificities of the dual-PCR test were 55% and 62% in patients with negative cytology and negative biopsy, respectively. Of thirty-six patients negative by both cytology and histology, 4 tested positive for HPV, giving an overall specificity of 88.9%. Water and unused napkin controls were uniformly negative, satisfying the requirement for zero cross-contamination.

**Table 2:** Results of HPV testing in various subsets of HPV-induced cervical lesions

Pathological diagnosis (n)	PG5+/6+ positive n (%)	MY09/11 positive n (%)	Positive by PG or MY n (%)

Negative cytology (42)	15 (36)	13 (31)	23 (55)
ASCUS (13)	6 (46)	9 (69)	9 (69)
LSIL (24)	11 (46)	13 (54)	21 (88)
HSIL (6)	4 (67)	5 (83)	6 (100)
Negative biopsy (21)	8 (38)	8 (38)	13 (62)
CIN 1 (60)	29 (48)	30 (50)	46 (77)
CIN 2 (3)	2 (67)	2 (67)	3 (100)
CIN 3 (12)	8 (67)	9 (75)	12 (100)
Microinvasive carcinoma (1)	0 (0)	1 (100)	1 (100)
Invasive squamous carcinoma (6)	3 (50)	6 (100)	6 (100)
Adenocarcinoma in-situ (2)	1 (50)	2 (100)	2 (100)
Endocervical adenocarcinoma (1)	0 (0)	1 (100)	1 (100)
Clear cell carcinoma (1)	0 (0)	1 (100)	1 (100)
Small cell carcinoma (1)	0 (0)	0 (0)	0 (0)

ASCUS, atypical squamous cell of undetermined significance; LSIL, low grade squamous intraepithelial lesion; HSIL, high grade squamous intraepithelial lesion; CIN 1, cervical intraepithelial neoplasia grade 1; CIN 2, cervical intraepithelial neoplasia grade 2; CIN 3, cervical intraepithelial neoplasia grade 3.

Typing of the amplimers revealed a variety of HPV types: 6, 16, 18, 31, 33, 39, 51, 52, 53, 56, 58, 61, 66, 68, 70, 71, 82 (MM4), 83 and CP6108 (high-risk and probable high-risk types are in bold; unknown risk in italics; remaining types are low-risk types) (23). HPV types identified in selected cases are listed in Table 3. HPV type 58 appears to be relatively common in this locality (24, 25).

**Table 3:** HPV types found in various cervical lesions by sequencing and flow through hybridization (HybriMax™)

Pathology	HPV type (sequencing)	Case No.

Squamous carcinoma	58, 6^a^	87
Squamous carcinoma	58	69
Squamous carcinoma	66	65
Adenocarcinoma	58	86
CIN 3	56	62
CIN 3	52	55
CIN 2	58	59
CIN 2	53	58
CIN 2	58, 52^a^	55
CIN 2	16	34
CIN 2	52	32

a HPV type identified by flow through hybridization (HybriMax™).

High-risk HPV types are in bold face. CIN 2, cervical intraepithelial neoplasia grade 2; CIN 3, cervical intraepithelial neoplasia grade 3.

Nine patients had HPV testing six weeks after LEEP (Table 4). Patients’ age range from 28-45 (average: 34.8). Three had CIN 2 and the rest had CIN 3. Three had positive resection margins. HPV testing on air-dried vaginal discharge collected 6 weeks post-operatively were negative in all nine cases by the dual-PCR tests. Follow-up of this subset of patients for an average duration of 24.3 months (range: 7-36 months) revealed no colposcopic or cytologic evidence of recurrence.

**Table 4:** Patient age, LEEP result and follow-up duration. All tested negative for HPV at 6 weeks

Age	Lesion	Endocervical gland involvement	Margin status	Last follow-up (month)

32	CIN 2	Not present	Negative	18
33	CIN 2	Present	Negative	7
30	CIN 2	Not present	Positive	24
39	CIN 3	Not present	Negative	22
28	CIN 3	Not present	Negative	33
31	CIN 3	Present	Negative	19
43	CIN 3	Present	Negative	33
32	CIN 3	Present	Positive	36
45	CIN 3	Extensive	Positive	27

CIN 2, cervical intraepithelial neoplasia grade 2; CIN 3, cervical intraepithelial neoplasia grade 3.

## DISCUSSION

Most other methods of cervical specimen procurement for HPV testing have assumed the need for preserved (in preservation solution) specimens. Others have emphasized the advantage of reflex cytology on liquid-preserved specimens. We favor air-drying of the specimens to enhance the ease of handling and compliance, having previously detected HPV in air-dried vaginal discharge (20). Here we showed that the invariable recovery of HPV from air-dried vaginal discharge of 26 patients with CIN2 or above provided evidence that this specimen is indeed satisfactory, especially for those with clinically significant lesions (CIN 2 or above). Because continued moisture and enzyme activity from contaminating neutrophils, bacteria and fungal organisms may compromise the quality of the specimen, we instructed patients to air-dry the specimens prior to mailing.

Although we did not systematically conduct opinion polls, patients have not expressed dissatisfaction with this method of specimen collection. In fact, the compliance was excellent. All recruited patients submitted the requested specimens. We have also demonstrated that it is feasible to receive specimens in the mail and that specimen processing can be done rapidly and without cross-contamination. In addition, we found that specimen storage in room temperature without preservatives or refrigeration is compatible with HPV detection even after several months (data not shown).

Our results showed that all 26 cases of CIN 2 or above, including 9 cases of various types of invasive carcinoma were positive for HPV DNA on PCR-testing of self-procured air-dried vaginal discharge. The importance of high-risk HPV in the pathogenesis of such lesions cannot be overemphasized (4, 26). The absolute sensitivity for HCL strongly suggests that this specimen type is robust and can be recommended for cervical cancer screening.

Some institutions have explored the role of HPV testing following surgical ablation of HCL (27, 28). We tested 9 premenopausal patients who had LEEP by the same method (Table 4) and found that HPV negativity six weeks after LEEP correlated well with favorable clinical outcome. The prognosis is not adversely affected by positive microscopic margins in the absence of HPV, as was the case in three of our patients. Our results agree with others that HPV testing should be offered to patients who underwent LEEP, as an assurance against disease recurrence.

The advantages of air-dried specimens are manifold. Patients can collect such specimens by themselves without the need for any special tools or the purchase of special equipments, which add to the cost, complexity, and stigmatization associated with it. Because the specimens can be mailed to the testing facility, the pre-analytic cost is further reduced. It also permits anonymity, thus facilitating the inclusion of as many women as possible – reporting can be performed on the internet if the patient self-assigns unique user identification. Finally, even women living in under-serviced localities may be extended the benefits of cervical cancer screening. This is particularly relevant to people living in rural areas in many continents, especially Asia, Africa, Australasia, and the Americas.

Our study is restricted by the referral nature of our clinic population. The true value of HPV testing in cervical cancer screening using our approach can only be realized in a population screening setting involving much larger number of subjects. In addition, other methods of HPV testing may be explored to enhance automation and further reduce the cost.

In conclusion, we demonstrated that self-procured air-dried vaginal discharge is a preferred specimen type for the identification of high-grade HPV-induced cervical lesions. Researchers can now focus on two issues, determining whether this approach to cervical cancer screening is acceptable to women and validating this approach as being applicable to the general population. Women who underwent LEEP for CIN and who did not recur were also negative for HPV, suggesting that eradication of CIN by LEEP also eradicates HPV. Like cutaneous wart, cervical HPV infection also seems to be a surgical disease. Resection or extirpation of HPV-associated cervical lesions might be employed more liberally to eradicate HCL as wells as HPV.
